# Accumulation of De-Icing Salt and Leaching in Spanish Soils Surrounding Roadways

**DOI:** 10.3390/ijerph14121498

**Published:** 2017-12-02

**Authors:** Esther Asensio, Víctor J. Ferreira, Gonzalo Gil, Tatiana García-Armingol, Ana M. López-Sabirón, Germán Ferreira

**Affiliations:** 1 Departamento Química Analítica, Universidad de Zaragoza, 50018 Zaragoza, Spain; estherac@unizar.es (E.A.); gonzalogil25@hotmail.com (G.G.); 2 Research Centre for Energy Resources and Consumption (CIRCE), CIRCE Building-Campus Río Ebro, Mariano Esquillor Gómez, 15, 50018 Zaragoza, Spain; tgarcia@fcirce.es (T.G.-A.); amlopezs@fcirce.es (A.M.L.-S.); gferreira@fcirce.es (G.F.)

**Keywords:** de-icing salt, roadway, saline-sodic soil, roadway, saline-sodic soil, plant grow yield, environmental impact, plant damage

## Abstract

The environmental implications of soil salinity caused by accumulation of de-icing salt and leaching in soils of northeastern Spain were examined. For this purpose, the concentrations of ions associated with diagnosing and managing this problem were evaluated from several measurements performed over one year along a road. This analysis demonstrated a higher concentration of soluble Na^+^ in the soil 3 m from a road in the northernmost part of the study area in February, which made the soil saline-sodic. Data from the rest of the study period (during the spring and summer) demonstrated that the de-icing salt moved to areas farther south by runoff water, which caused environmental impacts by modifying soil characteristics. These results suggest that leaching of Ca^2+^ and Mg^2+^ cations occurred faster in the studied systems in sodic soils. Leaching of these cations may affect plant yield, and results in environmental impacts within 3–30 m from the road. Awareness of this impact will be useful for developing future strategies for evaluating and reporting these complex relationships within Spain’s transport system and environment.

## 1. Introduction

During the cold winter period, large amounts of snow can be a problem for road traffic. De-icing salts are widely used to prevent the formation of ice on road surfaces, and scattering these salts on the road has been a practice carried out for many years in some countries. In the province of Huesca (Spain), during the winter of 2013/2014 alone, it was estimated that 900 km of roadways were affected by snowfall. In Spain, sodium chloride (NaCl) is the salt most commonly used by Direction General of Traffic (DGT) because of its ready availability, high effectiveness, and low cost. However, despite the benefits of solving the problem of ice on roadways, the indiscriminate use of massive amounts of thaw-salts can lead to severe environmental impacts, which have worried scientists for decades [1,2,3].

There is considerable evidence that salt accumulation affects aquatic ecosystems [4,5,6,7,8,9] and also damages terrestrial vegetation [10,11], but a great deal remains unknown about how far salt disperses overland from roads, how much is retained in soils before it passes on to plants, which mechanisms (e.g., meltwater runoff or airborne spray and dust) are most important in salt dispersal, and how it ultimately influences the plants. Therefore, knowledge of the distribution of salt concentrations in soils can help to estimate the impacts on the environments surrounding roads [12,13,14].

An increase in soil salinity can change soil conditions and, consequently, the suitability of the soil for plant growth, particularly in areas next to roads subjected to salt application in winter. Because of the action of Na^+^ from de-icing salt, which produces exchangeable sodium, leaching of ions such as Ca^2+^, Mg^2+^ and K^+^ occurs. Elevated sodium cation concentrations in soil tend to displace naturally occurring cations and disperse organic and inorganic particles in soil pores, which reduces soil permeability and aeration and increases overland flow, surface runoff, and erosion [15]. This phenomenon can seriously disrupt plant growth [16] and thus impact the environment.

In this regard, several soil parameters associated with soluble and exchangeable ion concentration, such as the Exchangeable Sodium Percentage (ESP), Sodium Adsorption Ratio (SAR) and salinity measured in terms of Electrical Conductivity (EC) (as well as others soil parameters like pH), are useful for characterisation of soils affected by de-icing salts. These parameters can help to measure changes in the soil microstructures, which can inhibit the functions of cell membranes, cause osmotic stress, decrease cell plasmolysis and chlorophyll levels. These effects result in disorders in photosynthesis, and thus adversely affect crop growth.

A study of soil characteristics as a result of de-icing salts was carried out in Spain as described herein. To the best of our knowledge, this is the first study of its kind in Spain. The study was divided into two parts. The first part consisted of characterisation of soils located along a 44 km segment of the road N-330 (Huesca, Spain), which was subject to the effects of the application de-icing salts. Experimental EC, SAR and ESP were calculated from soluble and exchangeable cation concentrations through characterisation techniques. The information obtained was analysed in terms of the relationship between the SAR and ESP of these soils. Most experimental SAR values obtained for all study points were <13, which showed a non-linear relationship between both parameters SAR and ESP, suggesting an outlier case. Therefore, the classification of soils was made using the parameters EC and ESP indicating non-saline-alkali soils in soils further north of the studied areas. These contain sodium in a quantity sufficient than can interfere with the growth of most plants, since soils characterized by having excessive levels of sodium that is able to displace others elements such as magnesium or calcium reducing, therefore, the levels of non-sodium based salts are lower. This phenomena can favour the degradation of the soil structure at the same time that it can cause to a decrease of the infiltration and permeability of the soil to water, leading to problems for plant growth [17]. Finally, the information obtained for these parameters (EC and ESP) was used to estimate environmental impacts and help to determine future strategies based on driving force-pressure-state-impact-response as a generic tool for reporting and understanding the complex relationships within the system of transport and the environment.

## 2. Experimental

### 2.1. Research Area and Soil Sampling

The research area is located in the province of Huesca, Spain, in the Pyrenees region. Monitoring was carried out in 2014 in the valley of Aragón, following the course of the Aragón River, which runs almost parallel to road N-330 from Astún to Jaca and N-240 toward Jaca from Pamplona. In detail, the soil investigated in the present study was next to the roads, and can be identified within reference soil groups as Haplic Phaeozems (Rioseta-Canfranc), Haplic Phaeozems association, Eutric Cambisols and Kastanozems (Villajuanita), and Haplic and Luvic Calcisols (Jaca).

It would have been interesting to monitor the salt used for de-icing roads across the entire province of Huesca where the surroundings may have been affected, but limited access led to selection of the abovementioned zones; therefore, the rest of the surrounding area was excluded from this work. For sampling, the research area was divided into three zones, with similar distances maintained between them (around 15 km). These zones were selected based on certain characteristics to provide the most representative sampling possible. Thus, the three zones had to be adjacent to the road, as flat and homogeneous as possible, and away from any barrier, guardrail, or other obstacles that could prevent the salt from spreading, or a surface sloping away from the road to ensure that further spread of salt would not be halted. These requirements had to be met for a large area to include the final sampling point 30 m from the road. The three zones selected based on these criteria are in Table 1.

**Figure 1:**
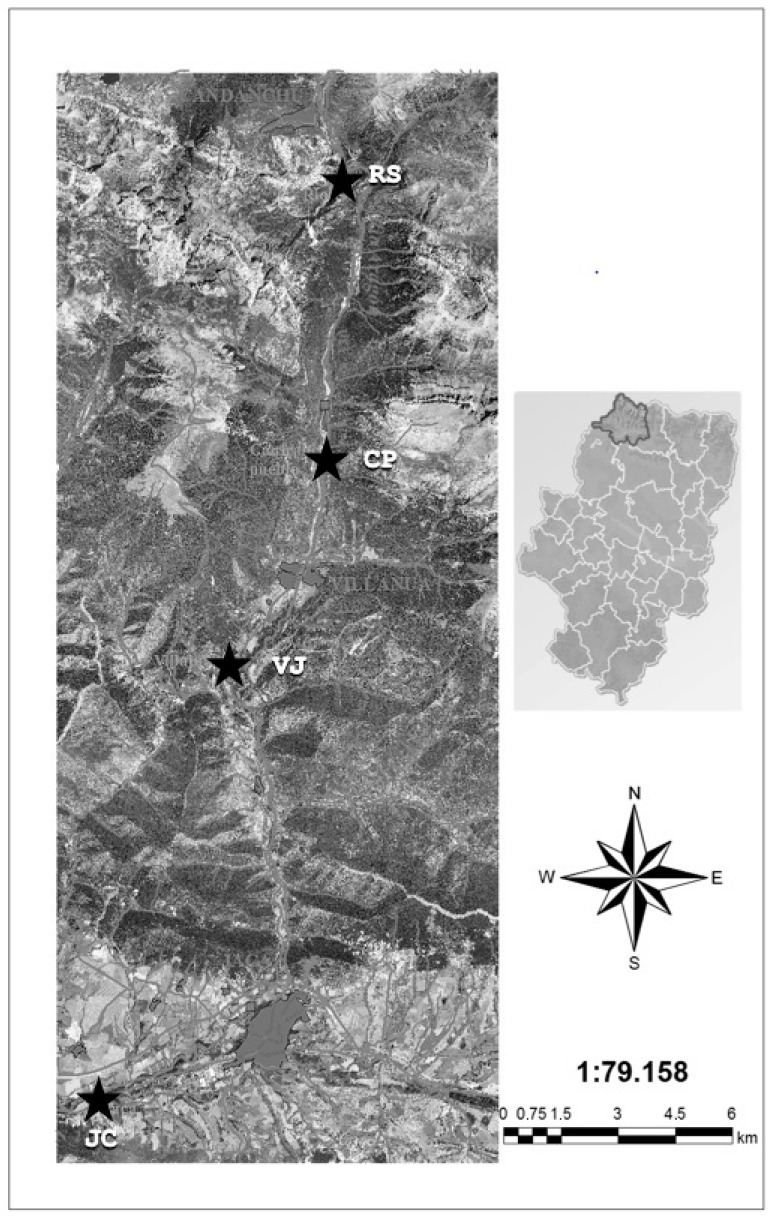
Sampling points.

Two sampling points were located in zone 1 because of the high salt dosage registered, which marked this zone as the most relevant study area. In contrast, zones 2 and 3 were areas with moderate and low dosages of salt, respectively. Zone 3 sometimes even exhibits a minimum contribution of de-icing salt, depending on how severe the winter has been. Zone 2 corresponds to a sampling point where there is no agricultural activity, while zone 3 is close to the Jaca urban zone. There, at least once a year the zone has agricultural activity. Figure 1 shows a map with the locations of the RS (Rioseta (sampling point)), CP (Canfranc (sampling point)), VJ (Villajuanita (sampling point)), and JC (Jaca (sampling point)).

Once the sampling zones were selected, to compare the involved ions before and after the salting period, soil samples were collected from January to September 2014. First, samples were collected during the winter (January and February), when de-icing salt application was most important. Then, samples were collected in spring (April–June), when the thaw and the mobilisation of saltwater took place, and finally in late summer (September) as a return to the reference point. In the first two periods, samples were collected twice. Additional sampling was not considered for interpretation of changing conditions because the above samplings were considered to be sufficient. The dates for sampling were as follows:

(1) Winter (application of de-icing salt): from January to February 2014.
(2) Spring (melting time): from April to June 2014.
(3) Late summer (control): September 2014.

The soil sampling plan followed in this study was based on the works of Bäcktröm et al. [18] and Lundmark and Olofsson [19]; at different distances from the road to identify possible relationships between the presence of the cation sodium and proximity to the road. According to these authors, distances are chosen based on the different ways by which sodium chloride can be mechanically transported after its application or other winter-maintenance actions. Briefly, the authors note that a considerable amount of de-icing salt can be present at 3 m with simple application by spreaders. Then, this salt can be moved up to 5 m by a snow remover. Next, the activity of dynamic snow, whereby snow is transported along with salt, can move salt up to 10 m. In theory, this distance would be the limit to which the NaCl would spread mechanically. Theoretically, NaCl should not be transported by mechanical actions to a distance of 30 m; therefore, this distance is used as control.

Thus, the soil was sampled along four parallel transects distributed evenly along the road section studied. Each transect had four sampling points located at 3, 5, 10 and 30 m from the road, except for the VJ point for which transects were at 5, 7, 10 and 30 m. For each sampling date, samples were taken from the four zones and transects mentioned above generating a total of 16 soil samples to analyse. Therefore, during the period of development of this work (5 months), a total of 80 soil samples were considered to be analysed.

Regarding the depth, a soil sampler with an auger to reach that depth was used to collect five subsamples comprising the upper 20 cm of soil at each sampling point. This value was considered to be enough based on studies found in literature. Amrhein et al. [20] mentioned that the concentration of ions added at the surface rarely increased below 20 cm, as long as mechanical processes were not involved. In addition, recent studies carried out by Nikolaeva et al. [21] analysed de-icing salts (DS, Cl^−^) in the topsoils (0–3 cm depth) sampled at distances of 1, 6, 10, 18 and 50 m perpendicular to the roadbed in three replicates 10 m apart. As conclusion, the topsoil is able to reflect the accumulation of de-icing salts. All subsamples from the same point were pooled into a composite sample before further analysis. In this way, around three kilograms of wet soil were collected, at each distance (0, 5, 10 and 30 m) and at each sampling point (RS, CP, VJ and JC), obtaining enough sample quantity to analyse and consider it representative of the sampling point.

### 2.2. Experimental Procedure

Soil samples were collected and put into suitable plastic bags for drying at room temperature on newspaper for a week. Once the samples were dried, gravel was removed using a 2-mm sieve. The final samples were stored in plastic bottles at room temperature until analysis.

#### 2.2.1. Technical Characterisation

All analytical parameters were determined in the laboratory, including pH, conductivity, and exchangeable cation concentrations. The determination of pH and conductivity was carried out by saturated paste extract with a soil-water ratio (1:2.5). The pH measurements were made with an model 920A pH apparatus (Orion Research Inc., Boston, MA, USA), whereas conductivity was measured with a conductivity meter, model 120 Microprocessor (Analytical Control Italia, S.p.A.r, Milano, Italy).

For the determination of soluble cations, the samples were prepared as described in UNE 77308:2001 [22] and then analysed using a SpectrAA 10 LX 800 spectrometer (Varian Australia PTF LTD, Mulgrave, Australia) which enabled determination of the cation concentrations of Na^+^, K^+^, Ca^2+^, and Mg^2+^.

For exchangeable determination, cation samples were subjected to a triple sequential extraction with ammonium acetate (1 M, pH 7) as an extractant. To ensure total removal, 5 g of dry soil was weighed and added to 33 mL of ammonium acetate (1 M). The mixture was covered and shaken for 5 min on a stir plate. The mixture was centrifuged for 5 min at approximately 2000 rpm. Once the solid phase was separated, the liquid supernatant was decanted into a 100 mL graduated flask. This process was repeated twice. The extracts were added to a volumetric flask, and distilled water was added to bring the volume to 100 mL. These samples were then analysed to determine the cation concentrations of Na^+^, K^+^, Ca^2+^, and Mg^2+^.

#### 2.2.2. Parameters Considered for Characterisation and Evaluation of the Effect of Salt on the Studied Soil

There are several pathways through which saline and/or sodic soils are generated, such as excessive application of fertiliser, manure or compost, or use of de-icing salts on sidewalks and roads. This process occurs because salts run off and enter the soil. This problem can be diagnosed by analysing the results obtained from measurements described in previous section. For this purpose, useful parameters associated with soil characteristics can help to elucidate the effects of de-icing salt on the soil. In particular, Electrical Conductivity (EC), the Sodium Adsorption Ratio (SAR) and the Exchangeable Sodium Percentage (ESP) are determined for this purpose, and are briefly defined below.

The first of these parameters, EC, indicates the salinity of the soil solution. In other words, it measures the ability of the soil solution to conduct electricity. This parameter is expressed in decisiemens per meter (dS/m, which is equivalent to mmhos/cm) [23].

Secondly, SAR is widely accepted as an index to characterise soil sodicity, which represents the proportion of sodium to calcium and magnesium in soil solution. This parameter can be calculated using Equation (1), where the concentrations of Na^+^, Ca^2+^, and Mg^2+^ cations are expressed in meq/L [23]:

(1) $$\mathrm{SAR}\;=\;\frac{{\mathrm{Ca}}^{2+}}{\sqrt{\frac{{\mathrm{Ca}}^{2+}{+\mathrm{Mg}}^{2+}}{2}}}$$

However, the last parameter, ESP, is another widely accepted index for characterising soil sodicity that can be determined using Equation (2) [23]:

(2) $$\mathrm{ESP}\;=\;\frac{{\mathrm{Na}}^{+}}{\mathrm{CEC}}\text{×}100$$

The Na^+^, Ca^2+^ and Mg^2+^ cations are considered dominant in this study; therefore, as an estimate of exchangeable hydrogen obtained from the buffer pH that makes up the total cation exchange capacity, the Cation Exchangeable Capacity (CEC) was calculated by summing the exchangeable concentrations of the predominant cations [Na^+^_x_], [Ca^2+^_x_] and [Mg^2+^_x_] in meq/100 g of soil (x denotes the exchange phase) [24].

## 3. Results and Discussion

### 3.1. Salinity of the Studied Soils

**Figure 2:**
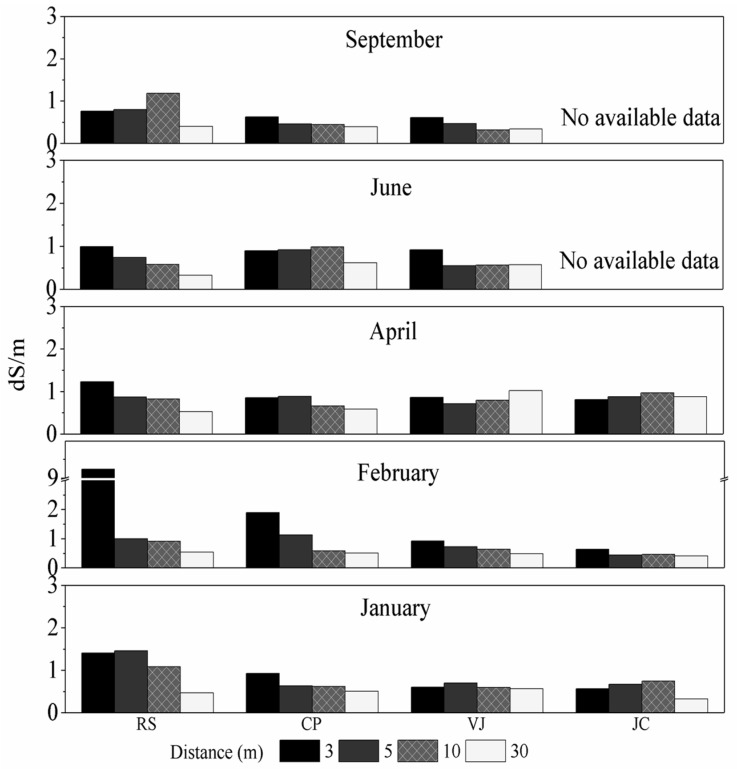
Profile of Electrical Conductivity (EC) for the studied zones during the study period and along the transects considered.

Figure 2 shows EC profiles for the studied zones during the study period and along transects considered. All EC values (except for February at 3 m) fall into a range between 0.0 and 4.0 dS/m, which indicates non-saline soil [23,24,25]. This finding indicates that from the viewpoint of soil salinity, most of the studied soils were non-saline along the profiles at distances from 3 to 30 m, except that single point in February. However, in general terms, it can be said that the EC decreased as distance from the road decreased for all studied zones. In contrast, significant differences were apparent for each transect over the studied period.

The RS case exhibited high EC values at 3 m in January and February. In fact, an extremely high EC value was noted at 3 m in February (around 9.2 dS/m). This value can be attributed to an enormous amount of salt visible to the naked eye beside the road forming a kind of wall at 3 m. Moreover, the EC values corresponding to the month of January at 3 and 5 m were higher compared to values for the farther distances of 10 and 30 m. For RS, EC at 3 m then decreased over the subsequent months to reach a value below 1.0 dS/m. A similar behaviour was also observed at 5 m.

For the point at a distance of 10 m at RS, EC also decreased, but only from January to June (1.1 to 0.5 dS/m), and then increased again to a value of 1.2 dS/m in September. This increment may be attributed to soluble Na^+^ coming from the area at a distance of 3 m where there was a large amount of salt. In general terms, EC decreased with increasing distance each month, with low values of EC at 30 m (around 0.5 dS/m) and the lowest value in September (around 0.33 dS/m).

**Figure 3:**
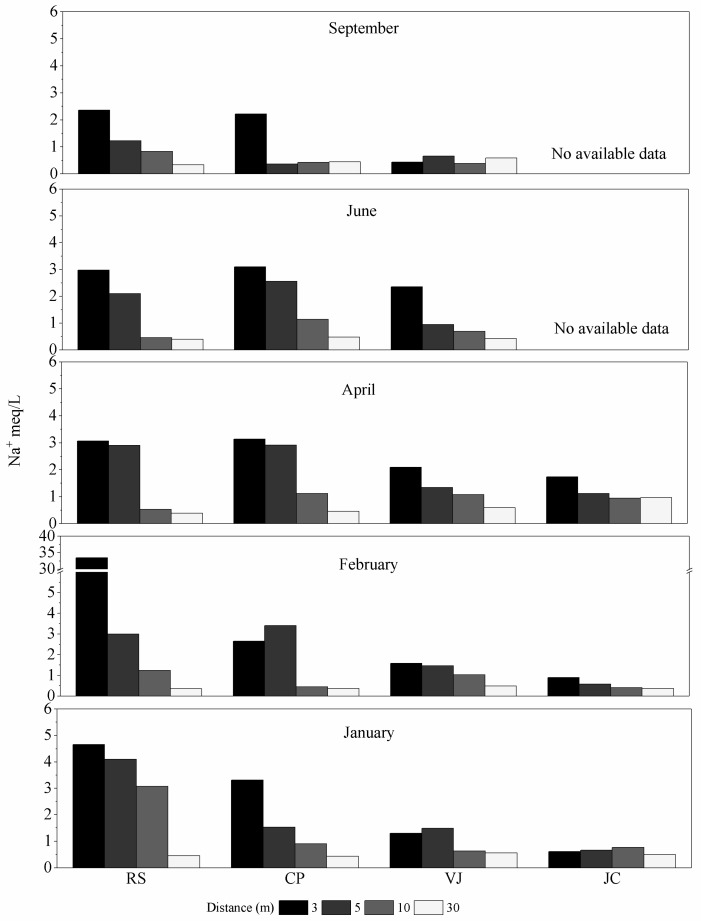
Sodium concentrations in soil solution.

Moreover, lower influence of de-icing salt was observed for the CP case, where lower EC values were measured in February relative to RS. Similarly, the EC decreased with distance from the road and with time. In contrast, significant differences were not observed for the VJ and JC zones; therefore, these results may be associated with soil composition and the seasonal development of soils. These findings can also be attributed to the geographical context of these zones, because they are located farther south where little salt was needed for de-icing. The above is corroborated by Figure 3, which shows the profiles of soluble Na^+^.

A high concentration of soluble Na^+^ was observed in February for RS (33.5 meq/L at 3 m). This result is consistent with EC at the same site in the same month. The trend for Na^+^ concentration is similar to the case of CP. The soluble Na^+^ concentration at 3 m remained almost constant (around 3.1 meq/L) from January to June, but then reached a value of around 2.3 meq/L in September. However, according to the sample results for each month, soluble Na^+^ concentration decreased with increasing distance, although this trend did not occur in February as the effect of the de-icing salt was still observed; that is, the soluble Na^+^ concentration in the soil increased to 3.5 meq/L at 5 m relative to 3 m. Then, it dropped drastically to 0.62 meq/L at 10 and 30 m. In conclusion, the CP site showed similar behaviour to the RS case, but with higher soluble Na^+^ concentration 3 and 5 m from the road, which may have been caused by the location directly receiving de-icing salt.

The VJ zone was further south with lower altitude; therefore, concentrations of soluble Na^+^ were below those obtained at the RS and CP sites (around 3.0 meq/L) at 3 and 5 m in the winter months (January and February). However, the soluble Na^+^ concentration was higher in April and June, and similar to CP, it dropped to lower values. Based on the geographical position of VJ, with less snowfall in the winter, the amount of sparse de-icing salt was lower; therefore, the sodium cation concentrations in the spring months likely came from water runoff from the road transporting salt that applied at higher elevations.

Finally, for the JC case, soluble Na^+^ concentrations could be determined only from January to April; sample collection was not carried out for the rest of the period because of technical incidents. However, compared with the other cases, a low concentration of soluble Na^+^ in the winter months was observed (<1 meq/L), although sodium reached higher values relative to the VJ case (April).

### 3.2. Sodicity of Studied Soils

As mentioned above, two parameters were evaluated for information about the soil sodicity: SAR and ESP. Both were analysed in this study to investigate the effects of de-icing salt on soils surrounding the roads. Here, this effect is addressed by classifying soils according to salinity and sodicity parameters, based on the scheme provided by the Natural Resources Conservation Service (NRCS) [23]. This classification scheme is shown in Table 2.

There is equivalence between SAR and ESP, which can be observed in Table 2. This fact is mathematically expressed by means of the relationship between SAR and ESP expressed through Equations (3) and (4) where ESR (exchangeable sodium ratio) refers to the exchangeable sodium ratio measured in the exchangeable phase, which is a linear function of SAR:

ESP = ESR (100)/(1 + ESR)(3)

ESR = −0.0126 + 0.01475 (SAR) (R^2^ = 0.852) (4)

Equation (4) was developed by the United States Department of Agriculture (1954) [26] and is one of several applications based on the Gapon equation. The latter was developed by Gapon in 1933 [27], based on a linear relationship between the ratios of ions (Na^+^ and Ca^2+^) in solution and the same ions in the exchange phase, as long as the ions are adjusted for valence. The U.S. Department based their study on a collection of 59 surface soil samples without consideration of differences in soil properties. The proportionality coefficient in the regression of ESR on SAR (0.01475 in the U.S. Department of Agriculture equation) corresponds to the exchange constant from the Gapon equation (K_G_).

Thus, the SAR and ESP values in Table 2 seem to be set by the U.S. Salinity Laboratory, and are somewhat arbitrary, although they are supported by many studies that indicate the ratio of cations in a soil solution reflects the ratio of cations of the corresponding adsorbed cations when the two phases are at equilibrium [28,29,30]. Nevertheless, Poonia and Talibudeen [31] reported that the soil properties are intrinsically related to the relative quantities of cations adsorbed, which in turn are reflected by the Na-Ca system. Then, Oster and Sposito [32] reported that when SAR is between 0 and 40, the estimation of ESP based on SAR does not differ greatly from that recorded in the USDA Handbook No. 60 [26]. Poonia and Talibudeen [31] found that surface density and organic matter content significantly affected Na-Ca exchange equilibrium. Thus, the exchange constant should be determined for soils that differ significantly in these properties [31].

Therefore, based on the studies by Poonia and Talibudeen [31], a regression analysis of SAR and ESR was performed in this study to find a specific relationship between both parameters for characterisation of the soils corresponding to the sites RS, CP, VJ, and JC. The obtained equations are shown in Table 3 and show a weak correlation between SAR and ESR, with Pearson’s coefficients much lower than the unit indicating a weak linear relationship.

This non-linear relationship was not expected because most experimental SAR values obtained for all study points were <13, within the range of 0–40; therefore, great differences between these parameters would not be expected. Thus, this result may suggest an outlier case that represents a set of soils with different behaviour from the soils studied by other authors.

In 1984, a similarly irregular case was studied by Frenkel a Alperovitch [33]. They reported divergences in the ESR and SAR relationship from that published by the US Salinity Laboratory. These authors studied 623 soil samples from arid and semiarid regions in Israel and found a relationship with the salinity and saturation percentage (SP). Their results revealed that a relationship between ESR on SAR does not exist with SP values between 20 and 40 or with low salinity levels (0.2–2.0 dS/m).

For this study, an approximate estimation of the SP was made based on mechanical constituents contained in the studied soils. That study is described in detail in Appendix (Appendix A.1) and it shows that the studied soils can have SP values between 20 and 40. Moreover, it can be seen from our results (Figure 2) that the soils studied in most transects have low salinity (EC < 2 dS/m), which corresponds to one of the possible requirements to explain the non-equivalence ESR on SAR in the range of 0 < ESP < 40.

Based on the above, the soils affected by de-icing salts were classified based on part of the common classification proposed in Table 2. In other words, only the parameters EC and ESP were taken into account. Two main classifications were made according to the results obtained. Namely, saline soils with EC of saturation paste extract higher than 4 dS/m and ESP < 15, and non-saline-alkali soils, which contain sodium in a quantity sufficient to interfere with the growth of most plants. The latter soils have ESP > 15 and EC of the saturation extract lower than 4 dS/m. In contrast, EC lower than 4.0 dS/m and ESP < 15, and EC over 4.0 dS/m with ESP over 15 correspond to a normal soil and a saline sodic soil, respectively [25].

**Figure 4:**
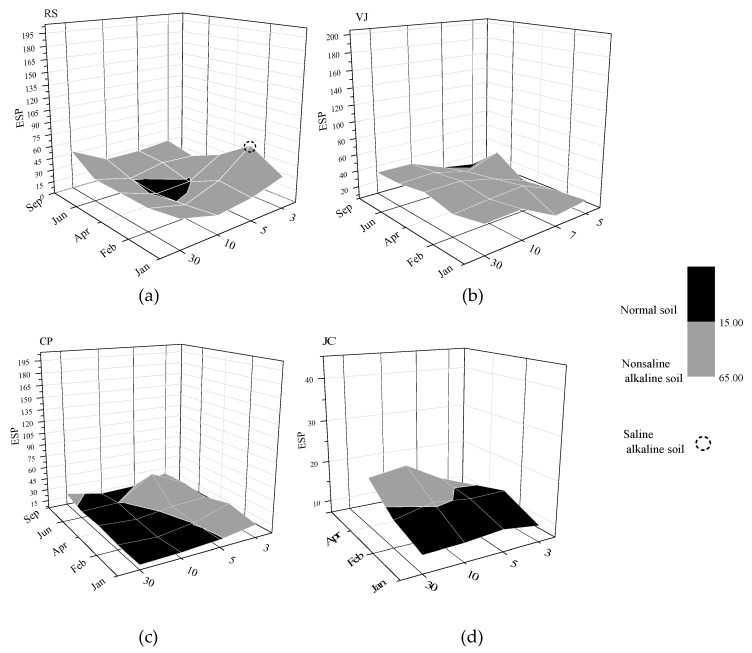
Soil sodicity: exchangeable sodium percentage (ESP) profiles of (**a**) Case RS (Rioseta (sampling point)); (**b**) Case VJ; (**c**) Case CP and (**d**) Case JC.

Figure 4a–d show the profiles of the soils affected by de-icing salt along 3–30 m transects for the studied zones. The zones most affected were RS and VJ, as indicated by ESP values higher than 15 during some of the studied months along transects studied, even comparing with data reported by the CSPS (around 3.3). In contrast, the RS case had non-saline alkaline soil during most of study period (January–September). However, saline alkaline soil was recorded in February 3 m from the road (with ESP and EC around 60 and 9.0 dS/m, respectively). This finding may be attributed to the fact that large amounts of de-icing salt were used in that month. Moreover, ESP at 3 and 5 m decreased over time (January–September) and achieved lower values of ESP, but remained above 15, whereas EC dropped below 2 dS/m (Figure 2). Nevertheless, ESP dropped dramatically below 15 at 10 m in April to generate a normal soil (see Figure 4a); then, ESP rose again in June and September, with high ESP values observed at 30 m. This behaviour can be attributed to the displacement of Na^+^ cations caused by movement farther from the road; consequently, it was more likely to remain in the pores of the soil clay. The possibility of accumulation of this cation from the previous year should also be taken into account.

A similar behaviour was observed in the VJ case (Figure 4b). Although values of ESP at 3 m could not be measured because of technical incidents, it was observed that ESP increased from 5 m up to 30 m for all months, which again reflects Ca^2+^ and Mg^2+^ cation leaching.

Regarding CP, despite the proximity between points RS and CP, which both belong to zone 1, ESP showed different behaviour in CP (Figure 4c) compared with RS (Figure 4a). The CP zone showed higher ESP than 15 up to approximately 5 m during the months studied. However, from 5 m, ESP dropped; a high ESP was only observed at 30 m in September, and it did not exceed 15. This result may be associated with the amount of de-icing salt applied on the road. Although zone 1 (RS and CP) was considered a potential area where a high salt dosage was registered, it may be that the RS point had a higher amount of de-icing salt compared with CP. Unfortunately, this inference could not be confirmed because the quantity of salt dispersed in the road close to each point was not controlled as part of this study, and it would difficult to determine this information.

Finally, although technical issues caused an interruption of the sampling procedure at the JC point during June and September, data for the start of the study period suggest that the soil of this point was less affected by de-icing salt, because ESP lower that 15 was observed throughout almost the entire studied period (Figure 4d). This result may be associated with the amount of de-icing salt used; as mentioned above, the zones 2 and 3 were subjected at moderate and low dosages of salt.

The above results may also be related to the organic matter present in the soil. The organic matter content of the study zones was investigated based on the Soil Property Databases of Spanish Soils (CSPS) report. An important difference was found in terms of organic matter content between RS and the zones farther south. Based on examination of a region close to the RS point, a percentage of organic matter around 13% was reported by the CSPS, whereas the other points farther south, close to zone 3 (JC), exhibited organic matter content between approximately 2% and 3%.

**Figure 5:**
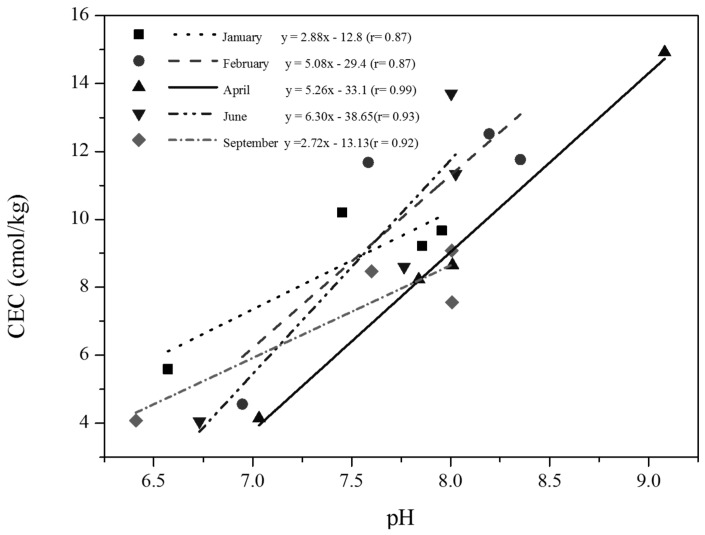
Cation exchangeable capacity (CEC) and RS-soil pH relationship.

The CEC helps to characterise the soil types under consideration through ESP, because CEC is in turn related to negative charge. Permanent negative charge is located within the structure of clay particles, whereas variable charge is located on the edges of clay and organic matter particles. Therefore, the CEC used for ESP calculation, measured in a laboratory, indicates the ability of a soil to hold cations against leaching [25]. Moreover, a linear relationship was found between the CEC and pH along transects of the RS zone in each month of the studied period (Figure 5). Thus, despite of the low organic matter content, it may be possible that there is enough variable negative charge in the RS zone. The primary factor affecting variable charge is pH; high pH increases CEC. In fact, soil CEC can be expected to increase by up to 50% if the pH is changed from 4.0 to 6.5, and to nearly double if the pH is increased from 4.0 to 8.0 [31]. Thus, the relationship between CEC and pH can help to explain the variability of the amount of organic matter in zone 1 between RS and CP.

In conclusion, the marked differences in organic matter content between the points and the amount of de-icing salt added along these zones can help explain the differences of ESP observed, with higher values for RS compared with CP and JC.

### 3.3. Environmental Implications

The immediate impact of the de-icing salt is reflected by the vegetation adjacent to roads, which can be subjected to environmental stress impairing its natural balance. Plants are known to need energy tolerate or resist the potential stress caused by de-icing salt use (see Appendix A.2.1). This impact can be measured as a reduction in plant growth. Additionally, according to Hanes et al. [34], one of the factors affecting plant growth is osmotic pressure, which plays an important role for plants because they take in water from the surrounding soil through their roots (see Appendix A.2.2). Although water is naturally attracted to ions in salt concentrations, an increase in soil salinity changes the osmotic pressure gradient, making more difficult for plants to take in water.

Apart from affecting water availability, a high-EC (High Salinity) environment also reduces the availability of essential plant nutrients. These factors cause a reduction in plant yields, and induce extreme damage in many cases. Moreover, high ESP (sodicity) also considerably influences on plant yields. Therefore, these two parameters (EC and ESP) can be represented by the environmental impact reflected as a decline in plant growth.

Based on above, this study relies on the estimation of potential damage to the vegetation surrounding the above zones affected by the de-icing salts based of the measured parameters EC and ESP. It is made by combining the general crop response as a function of EC and reduction in crop yield as a function of soil ESP. Thus, the environmental effect of de-icing salt on the vegetation surrounding the roads of the RS and CP (1), VJ (2) and JC (3) zones, can be characterised by identifying ESP and EC for a set of points through plotting. As a result, different sectors can be identified as a function of the average percentage of reduction in plant growth depending on salinity (EC) and sodicity (ESP).

**Figure 6:**
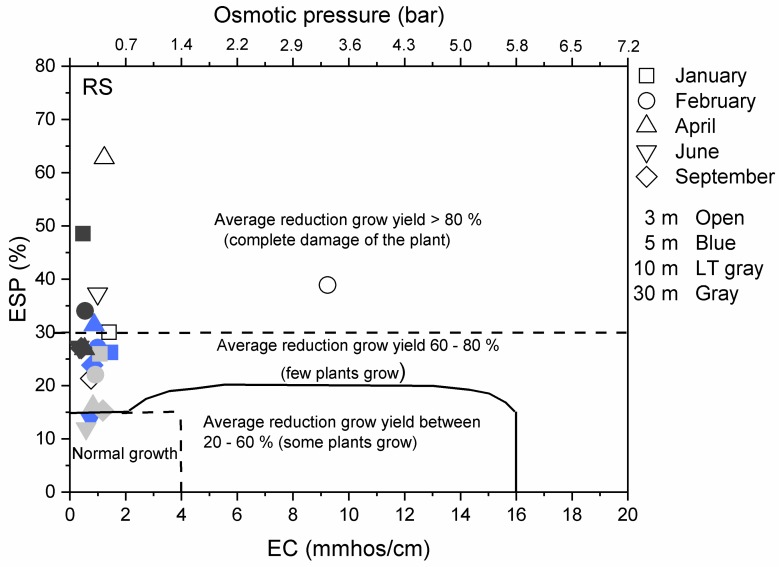
Overall environmental impact of de-icing salt on the vegetation surrounding the road in the RS zone.

In other words, the overall environmental of exposure of the vegetation to de-icing salt effects can be placed in ESP vs EC zones with different percentages of average reduction grow yield. During the studied period, most of transects exhibited conditions with high probabilities to have an overall reduction in growth yield, and, consequently, low plant yield. In detail, the results for the RS case are shown in Figure 6. Transects at 10 and 30 m in June and September showed possibilities to meet conditions for a normal growth after salt spreading.

This finding can likely be attributed to the effect of seasonality, and the rainy period in particular. In contrast, one transect (at 3 m from road) exhibited the worst conditions with complete damage to the plants. First, in February, the results are consistent with the saline-sodic soil obtained in the previous section. Then, at the same 3 m distance in April, it likely was caused by salt remaining in the environment. A similar analysis has been performed for the other locations selected for the study. Further explanations can be found in Appendix A.2.3.

The above results provide an idea of the severe effects of de-icing salt on the vegetation surrounding the road in this area of Spain. Under the complex mechanisms of de-icing salt damage, vegetation between 3 and 30 m from roads is subjected to serious environmental impacts, which is consistent with the work of Fan et al. [35]. These authors addressed potential mechanisms based on aerial deposition and soil uptake and showed potential effects of soil uptake of salt extending at least 100 m from roads.

## 4. Conclusions

The effects of de-icing salt on Spanish soils were investigated based on key parameters (EC, SAR and ESP). In addition, the potential environmental impact was quantified through EC (salinity) and ESP (sodicity) based on a general level of crop tolerance to salt indicating no effect on the plant yields.

The results showed higher soluble Na^+^ concentration in the RS zone 3 m from road in February, over 10 times higher when compared with other zones. Similarly, high EC was observed in the same month at 3 m, which together made the soil saline and sodic. Moreover, the zones that received higher salt dosages (RS, CP and CJ) exhibited sodic soils with SAR < 13 (no relationship was found between ESR and SAR).

Except a point at the RS site 3 m from the road in February, the environmental impact on the vegetation around the road (within 3–30 m) in the RS, CP and VJ zones resulted in a possible average reduction in growth yield of 60–80%. The RS case at 3 m (February) showed high-salt conditions for complete damage to the plants (average reduction in growth yield > 80%).

In general terms, these results suggest that fewer plants grow surrounding the roads because of the effects of de-icing, but further studies should be carried out to quantify vegetation damage caused by de-icing salts to represent all conditions and the nature of native plants in this region.

## Figures and Tables

**Table 1:** Sampling zones with their location coordinates.

Zone	Length (km)	Sampling Points	UTM Coordinates
1	14.9	Rioseta (RS)	Huso 30 X: 7013.53 m; Y: 4,738,572.32 m; Z: 1399 m
		Canfranc (CP)	Huso 30 X: 702,476.7 m; Y: 4,730,979.37 m; Z: 1004 m
2	13.8	Villajuanita (VJ)	Huso 30 X: 700,127.46 m; Y: 4,725,426.96 m; Z: 922 m
3	14.9	Jaca (JC)	Huso 30 X: 696,368.79 m; Y: 4,714,147.81 m; Z: 724 m

**Table 2:** Soil classification of salt-affected soil [23].

Class of Soil	EC (dS/m)	SAR	ESP
Normal	Below 4.0	Below 13	Below 15
Saline	Above 4.0	Below 13	Below 15
Sodic	Below 4.0	Above 13	Above 15
Saline-sodic	Above 4.0	Above 13	Above 15

SAR: Sodium Adsorption Ratio; ESP: Exchangeable Sodium Percentage.

**Table 3:** Regression equations for ESR (exchangeable sodium ratio) vs. SAR.

Point (Zone)	Equation	R^2^
RS	ESR = 0.0354 + 0.0225 (SAR)	0.18
CP	ESR = 0.0104 + 0.1952 (SAR)	0.04
VJ	ESR = 0.6641 − 0.2179 (SAR)	0.42
JC	ESR = 0.1027 + 0.043 (SAR)	0.43

CP: Canfranc (sampling point); VJ: Villajuanita (sampling point); JC: Jaca (sampling point).

**Figure A1:**
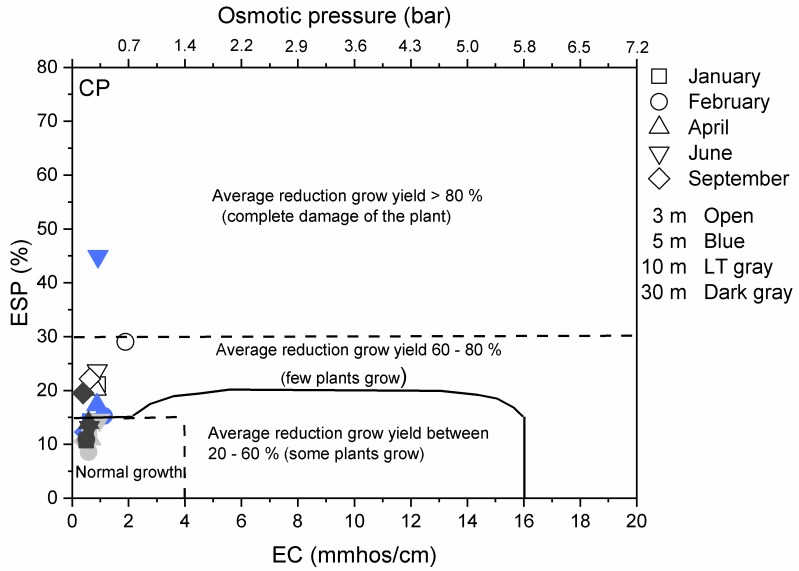
Overall environmental impact of de-icing salt on the vegetation surrounding the road in the CP.

**Figure A2:**
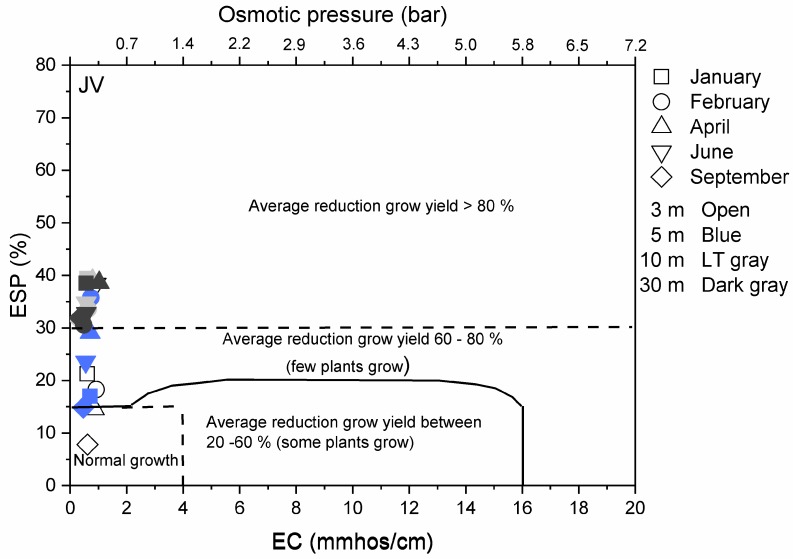
Overall environmental impact of de-icing salt on the vegetation surrounding the road in the VJ.

**Figure A3:**
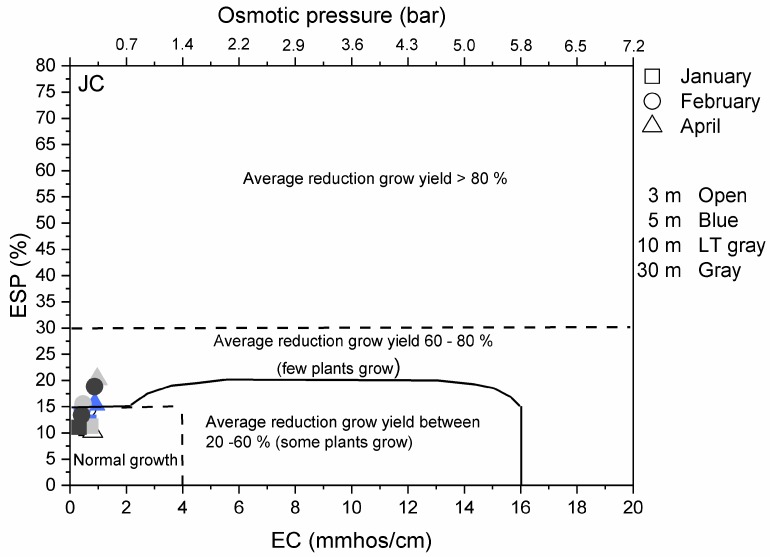
Overall environmental impact of de-icing salt on the vegetation surrounding the road in the JC zone.

## Abbreviations

The following abbreviations are used in this manuscript:

CEC: Cation Exchangeable Capacity
CP: Canfranc (sampling point)
CSPS: Soil Property Databases of Spanish Soils
DGT: Direction General of Traffic of Spain
DPSIR: Force-pressure-state-impact-response
DS: De-icing salts
EC: Electrical Conductivity (measurement of salinity)
EEA: European Environmental Agency
ESP: Exchangeable Sodium Percentage (measurement of sodicity)
ESR: Exchangeable Sodium Ratio
JC: Jaca (Sampling Point)
NRCS: Natural Resources Conservation Service
LT gray: Light gray
RS: Rioseta (sampling point)
SAR: Sodium Adsorption Ratio
SP: Saturation Percentage
UTM: Universal Transverse Mercator coordinate system
VJ: Villajuanita (sampling point)

## Appendix A

### Appendix A.1. Estimation of Saturation Percentage (SP) of Studied Soils

An approximate estimation can be made based on mechanical constituents contained in the studied soils. Such properties are Clay%, Silt%, and Sand%, which can be used in Equation (5) proposed by Stiven and Khan [36]:

SP = 578.72 − 4.54 (clay%) − 5.40 (silt%) − 5.56 (sand%) (5)

Thus, although determining the SP was not an objective of this study, an estimation of SP using these mechanical constituents could be carried out for several horizons based on the constituents tabulated in the Soil Property Databases of Spanish Soils (CSPS) [37]. To this end, an average of values in the environs of the zones under study was determined. The results are summarised in Table A1.

**Table A1:** Properties of the studied soils.

Point (zone)	Clay Average (%)	Silt Average (%)	Sand Average (%)	SP ± 6.89
RS and CP	25	38	37	48
VJ and JC	16	11	70	57

SP: Saturation Percentage.

It is important to note that the values in Table A1 do not precisely represent the studied zones. In fact, according to the data in the CSPS report, the data for mechanical constituents attributed to zone 1 (RS and CP) correspond to a region relatively close to zone 1. In contrast, in the case of zones 2 and 3, the average values were taken from a region a bit farther away. Therefore, the proximity of the studied zones to those assumed from the CSPS allows estimation of SP, as shown in Table A1. Thus, considering the deviation in the parameters (SP ± 6.89), the minimum value of SP (close to 40) for RS and CP (zone 1) may represent the upper limit value of the SP range (20–40) reported by these authors. Therefore, there is a possibility of a lower SP value than 40; however, further experimental studies must be performed to corroborate this finding.

Nevertheless, the CEC can also help to corroborate the low SP of the studied soils. This parameter has been reported as a measure of soil texture that allows estimation of water-holding capacity and cation exchange capacity.In fact, Sibbet et al. [25] reported a relationship between the SP, soil texture, and CEC in which soil texture corresponds to sandy loam exhibiting values of CEC in the range 7–15 meq/100 g and a SP between 20 and 35. In our study, all data for the RS, CP, VJ, and JC points showed a range of CEC between 6–12 meq/100 g. This finding may suggest that the studied soils have low SP because their CEC values are almost in the range of 7–15 meq/100 g.

In this light, these results may indicate that the set of soils from RS and CP (zone 1) points present characteristics like those studied by Frenkel and Alperovitch [28], which generated a peculiar behaviour where ESR does not have a relationship with SAR; that is, cations in soil solution do not reflect the ratio of cations of the corresponding adsorbed cations. This interesting issue will motivate further studies regarding these soils of Aragon. Concerning the VJ (zone 2) and JC (zone 3) zones, according to the average value of SP from the CSPS report (SP = 52), the value of SP would be found outside the range of 20-40; that is, above the upper limit. However, the values of CE were also below 2 dS/m; therefore, the assumptions made for zone 1 may reasonably be applied to zones 2 and 3.

### Appendix A.2. Environmental Implications

#### Appendix A.2.1. Main Factors Affecting Plant Growth

The immediate impact of the de-icing salt is reflected by the vegetation adjacent to roads, which can be subjected to environmental stress that impairs natural balance. According to Hanes et al. [34], there are three factors whereby high salt concentrations affect plant growth. The first is osmotic pressure, which plays an important role for plants because they take in water from the surrounding soil through their roots. Although water is naturally attracted to ions in salt concentrations, an increase in soil salinity changes the osmotic pressure gradient, and thus makes it more difficult for plants to take in water. The second factor is accumulation of salts in plant tissues, and the third is ionic imbalance causing desiccation and leaf-burn.

The latter is associated with the high amounts of sodium relative to calcium and magnesium ions absorbed in clays, which affect the individualisation and dispersion of the particles (in alkaline or sodic soil). This phenomenon can be explained based on the double-layer theory of Gouy-Chapan [38]. The clay particles offset the negative charge being surrounded by cations, which are subjected to two opposite strengths. The first strength is an attraction electrostatic force, and the second strength is diffusion; both are from surface of the clay where the cations are exchanged toward the solutions of the soil where there is a low cationic concentration.

The above consequences can be quantified based on two parameters described and discussed in the previous section as a way to characterise the implications from an environmental standpoint associated with the vegetation surrounding the road up to 30 m from road along 44 km of, across several zones at different altitudes. Understanding the effects of salt based on these parameters can help greatly to obtain an environmental metric focused on vegetation damage caused by traffic activities associated with the use of this kind of salt for de-icing in Spain. The above evaluation can be based on one of the pioneering countries in this field, Sweden, where advancements in this issue have led to proposal of indicators for monitoring the system of de-icing salt use and its impacts. This monitoring is achieved by a pertinent entity in charge of follow-up on how de-icing salt use affects the environment in various ways. This strategy is based on the environment and safety of the road system, and with other national entities, a follow-up system of environmental and traffic-safety features has been formulated for the road transport system [39].

#### Appendix A.2.2. Force-PRESSURE-State-Impact-Response (DPSIR) Approach

In 2001, Blomqvist [40], suggested a causal chain linking the activity of de-icing actions to damage to vegetation using the driving Force-pressure-state-impact-response (DPSIR) approach. This model connects different environmental problems, their causes, and society’s responses in an integrated way. In fact, the European Environmental Agency (EEA) uses the DPSIR approach as a generic tool for understanding and reporting the complex relationships within the transport system of and the environment. This system is well described by Blomqvist [40,41] as a de-icing and damage to vegetation in rural areas.

Based on this perspective, five indicators for de-icing effects are defined as D (driving forces); P (pressure); S (state); I (impact) and R (response). Of course, if this system model was considered, a great deal of work would need to be carried out related to indicator quantification based on representative and standardised parameters for further comparison. This integrated system is beyond the scope of this study, but for future strategies, this study provides a significant contribution as a starting point, because it addresses a portion of the descriptive impact indicators, the potential stress caused by the de-icing salt use; that is, the impact of vegetation exposure to the effects of salt.

#### Appendix A.2.3. Results on Overall Environmental Impact of De-Icing Salt on the Vegetation Surrounding the Road

The overall environmental impacts of exposure of the vegetation to de-icing salt effects for de different case studies are shown in Figure A1, Figure A2 and Figure A3.

Zone 1 location (CP) shown in Figure A2 is similar to RS (show in Figure 6 within the main text of the present paper), but more sample transects of CP (around 60%) are in the normal range. In contrast, less 40% of transects are in the range where few plants grow. Most were in January when large amounts of salt were spread, but not as much as in the RS zone.

Regarding the VJ zone (Figure A2), the transects show similar profiles to those of the RS zone, but the transects of VJ (3–10 m) during the winter period are close to the normal growth area, and even the point at 30 m is in this area in January, which means weaker effects of de-icing salt because of the low amounts spread in this zone. However, during the spring and summer months, plants in the VJ zone (3 to 30 m from the road) were subject to strongly damaging conditions. This finding can be attributed to runoff from the road transporting salt applied at higher areas (such as RS and CP).

Finally, in the case of JC (Figure A3), 83% of transects were in or very close to the normal zone. Although measurements were not possible during June and Septembers, values in this zone for average reduction grow yield by 60–80% were observed in February and April. This finding indicates that transects farther from the road could be subjected to the effects of de-icing salts transported by runoff and salt applied to the north.
